# Effects of Sorghum Malting on Colour, Major Classes of Phenolics and Individual Anthocyanins

**DOI:** 10.3390/molecules22101713

**Published:** 2017-10-12

**Authors:** Ali Khoddami, Mohammad Mohammadrezaei, Thomas H. Roberts

**Affiliations:** 1 Plant Breeding Institute, Sydney Institute of Agriculture, University of Sydney, Sydney, NSW 2006, Australia; thomas.roberts@sydney.edu.au; 2 Young Researchers and Elite Club, Isfahan (Khorasgan) Branch, Islamic Azad University, Isfahan 81595-158, Iran; mmohammadrezaee@uma.ac.ir

**Keywords:** sorghum, malting, phenolic compounds, 3-deoxyanthocyanins

## Abstract

Sorghum (*Sorghum bicolor*) grain contains many health-promoting phytochemicals, including a broad range of phenolic compounds. Malting of cereal grains is known to increase the bioavailability of macro- and micronutrients. However, the detailed effects of malting on sorghum grain anthocyanins, a major class of phenolics that influence the taste and colour of sorghum-based foods, requires further investigation. Eight commercial sorghum hybrids harvested from three regions in eastern Australia were malted and analysed for colour, tannin content, total phenolic content (TPC), flavan-4-ols, total flavonoids, total anthocyanins and 3-deoxyanthocyanins. Grains of all the sorghums were found to be tannin-free. Malting decreased the TPC of all samples. For TPC, the grand means among all the sorghum cultivars for raw and malted grain were 2.77 and 2.48 mg gallic acid equivalents (GAE)/g, respectively. For flavan-4-ols, the grand means for raw and malted sorghum grains were 2.98 and 2.23 abs/mL/g, respectively. Remarkably, total anthocyanin levels more than doubled upon malting whereas total flavonoid levels decreased by 12%. The average abundance of 3-deoxyanthocyanins in raw sorghum grains increased for about 8-fold upon malting. Our results will be valuable for sorghum breeders in the selection of lines for specific end uses and for food scientists developing sorghum-based products.

## 1. Introduction

Sorghum is ranked globally as the fifth most important cereal crop after wheat, rice, maize and barley, with a world production in 2016 of 812 thousand tonnes of grain [1]. Sorghum grain is a staple food for about 7% of the world population in 30 countries in Africa and Asia as well as a fodder and feed crop for livestock in 105 countries [2]. Grain sorghum production is the third leading cereal crop in Australia behind wheat and barley [3], and is equivalent to 3.25% of world production [1]. In Australia, most sorghum is grown in the eastern states of Queensland (Qld) and New South Wales (NSW).

Only a small portion of world total sorghum production is used for human consumption. However, in Africa and Asia, whole sorghum grain, decorticated grain or sorghum flour are commonly used to make foods such as thin or thick fermented or unfermented porridge, boiled products similar to those made with maize or rice grits, popped sorghum, flat bread and various types of deep-fried food. Sorghum has been used in the production of beverages such as non-alcoholic fermented beverages and (alcoholic) beer in Africa and Mexico, as well as spirit and vinegar in China. Sorghum is also used as a wheat substitute in gluten-free foods [4].

The chemo-protective nature of phytochemicals and limitations on the addition of synthetic antioxidants to food have attracted attention worldwide on phytochemicals as good sources of antioxidants and food/beverage colourants [5,6,7]. The phenolic compounds of sorghum are well known to act as antioxidants in vitro [8] and some are more potent antioxidants than vitamins found in other plant species [9]. Sorghum phytochemicals, particularly anthocyanins and other flavonoids, appear to exhibit anti-inflammatory [10,11], antidiabetic [12,13] anticancer [13,14,15,16], antilipidemic [17], immunomodulatory [17], antianaemic [18], neuroprotective [19], antidiarrhoeal [20], antimicrobial [21], and anthelminthic [22] activities.

Malting can be defined as the process of steeping, germination and drying (kilning) of cereal grains to advance the production of hydrolytic enzymes (responsible for converting starch into simple sugars and other hydrolytic activities), which are absent in ungerminated grains [23]. Malting has an impact on the abundance and profile of phytochemicals in sorghum grain, which in turn has an influence on the potential health effects of the finished product. Mixed results have been obtained for the effects of malting on the abundance of phenolics in sorghum grain, although most studies have found a decrease [24,25,26,27,28,29,30,31,32,33]. Elmaki et al. [26] reported a significant decrease in tannin content during germination. Osuntogun et al. [29] also found that the total polyphenol and tannin content of different sorghum varieties decreased during malting. In contrast, other workers observed an increase in polyphenols during sorghum germination, including total phenols, tannins, total leucoanthocyanin and total anthocyanin [32]. Ahmed et al. [31] also found an increase in tannin content in sorghum upon malting.

Almost all of the research conducted on the effect of malting on phenolics has been conducted on sorghums grown in Africa (Nigeria, Sudan, Benin) and has covered total phenolic content, tannin content and total abundances for specific classes of polyphenols such as anthocyanins. There is a lack of research on the levels of grain phenolics in Australian hybrid sorghums and the effects of malting. Therefore, in comparisons among grains of eight Australian sorghum hybrids harvested from three regions in NSW and Qld, the objectives of this study were:

1. To determine the effects of malting on the abundance of total phenolics, total flavonoid content, flavan-4-ols, total anthocyanins, as well as on colour;
2. To characterize and quantify the effects of malting on 3-deoxyanthocyanins.

## 2. Results and Discussion

### 2.1. Evaluation of Colour

The mean L*, a* and b* values for raw sorghum grains across the sorghums tested were 43.26, 16.77 and 16.99, respectively, while the mean values for malted grains were significantly lower (*p <* 0.05) at 40.82, 15.10 and 14.44, respectively (Table 1). Comparing growing regions, the mean L*, a* and b* values were significantly different (*p <* 0.05) and sorghum grain grown at Norwin had the highest level of reddish colour and lowest level of lightness.

The effects of malting on the grain colour of individual sorghum cultivars are shown in Table 2. Malted grains had lower L*, a* and b* values than raw sorghum with the exception of the a* value in G99. Buster had the highest lightness (L* value) while raw G22 had the highest redness and yellowness (a* and b* values).

### 2.2. Evaluation of Tannin Content

Tannin is a powerful antioxidant but the anti-nutritive properties of condensed tannin are undeniable [34]. The primary limiting factor in the use of sorghum grain in food products is the permanent chelation of proteins by tannins and anthocyanidins. A secondary factor is the significant reduction of starch digestion by binding of these phenolics to carbohydrates [35,36]. The Clorox bleach test for a pigmented testa is a straightforward test for the presence of condensed tannin in sorghum; sorghums lacking a pigment testa are considered to be ‘tannin-free’ [37]. A pigmented testa was found to be absent in all eight cultivars harvested from the three different regions, which indicated that they were type I sorghums (lacking condensed tannin).

Vanillin-HCl assay results gave the same pattern of results as the bleach test. In this assay, phenolic compounds other than condensed tannins may react with the vanillin reagent [38]. An overall analysis of the effects of malting on ‘tannin content’ (based on the vanillin-HCl assay) gave a 26% decrease for malted grain compared to raw sorghum (Table 3).

This might be due to phenolics leaching out of the grain during soaking and germination [24]. Grains from Yallaroi and Norwin regions showed significantly higher levels of tannin than Bellatta (*p <* 0.05) (Table 3).

For individual cultivars, the highest levels of condensed tannin in raw and malted grain were 1.02 and 0.77 mg CE/g for G22 and Eclipse and the lowest were 0.69 and 0.55 mg CE/g for raw Buster and malted G99 and Buster, respectively (Table 4). As the bleach test results for all the sorghum grains were negative, the low tannin values observed in the vanillin-HCl assay may have been due to interference from other non-tannin phenolics with the reagents [38]. The tannin contents of all Australian sorghum cultivars obtained here were in agreement with that of type I sorghums in the literature, which are reported to be between 0 and 3.80 mg CE/g [39,40,41].

### 2.3. Evaluation of Total Phenolic Content (TPC)

The grand means for TPC in the raw and malted grain samples of all the sorghum cultivars combined were 2.77 and 2.48 mg GAE/g, respectively (Table 3). Thus, TPC decreased almost 10% upon malting (*p <* 0.05). Grains from Yallaroi and Norwin regions showed significantly higher levels of TPC than Bellatta (*p <* 0.05).

Table 4 shows TPC for each of the individual raw and malted sorghum grain cultivars. Malting decreased the level of TPC in all samples, with the highest TPC for raw Eclipse (2.97 mg GAE/g) and the lowest TPC (2.21 mg GAE/g) for malted Buster. Yang [42], Dykes [43] and Njongmeta [44] reported that TPC of non-tannin raw sorghums was 0.9–18.2, 1.8–6.0 and 2.3–5.6 mg GAE/g while tannin sorghums had 5.1–29.6, 8–20 and 3.4–14 mg GAE/g, respectively. The results obtained in the present study for raw red Australian sorghums were at the low end of the range for non-tannin sorghums reported earlier.

The results here on the effect of malting on sorghum grain TPC were in agreement with reported decreases in phenolics after malting [26,29,45,46,47]. Ramadan et al. [48] also reported a decrease in total phenolics in sorghum, wheat and corn after soaking and germination. Our results were in contrast to the reported values for TPC in sorghum after malting by Nwanguma and Eze [32], which showed an increase in TPC after malting. Dicko et al. [49] reported no general impact of germination on sorghum TPC. These apparent differences in response of TPC could be due to the details of the different stages of the malting process (soaking, germination and kilning or their combination) applied to the sorghum grain. Applying a low kilning temperature (48 °C), for instance, would be expected to result in less decomposition of heat-labile phenolics than the commercial kilning temperature applied here (up to 85 °C).

The variation of TPC in raw and malted sorghum cultivars could be due to a number of factors such as genotype, growing location and growing system [39]. The decrease in TPC upon malting might be due to leaching of polyphenols into the soaking medium during the malting process. It is also likely that some of the polyphenols may enter into the endosperm with the imbibition water during germination, resulting in a decrease in TPC [26,46,50].

The kilning stage of the malting process performed in the present study might have led to phenolics forming insoluble complexes (via hydroxyl groups) with proteins, carbohydrates and minerals, or might have caused heat-induced polymerization or degradation, leading to a decrease in the apparent concentration of phenolics [27,51].

### 2.4. Evaluation of Flavan-4-ols

Flavan-4-ols (apiforol and luteoforol) are colourless phenolics produced from flavanones. Sorghums containing higher levels of flavan-4-ols have been shown to be more resistant to mould and are more drought tolerant [52,53]. Flavan-4-ols also may act as antioxidants and therefore have health benefits [39,54,55].

The grand means for flavan-4-ol content in the raw and malted sorghums were 2.98 and 2.23 abs/mL/g, respectively (Table 3). On average, malting significantly reduced the content of flavan-4-ols by 25% (*p <* 0.05). An overall comparison of the growing regions indicated that sorghum harvested from Bellatta had a significantly greater level of flavan-4-ols (2.70 abs/mL/g) than the grains harvested from Yallaroi and Norwin (2.57 and 2.54 abs/mL/g).

The flavan-4-ol contents of raw and malted grains for each of the individual sorghum cultivars are presented in Table 4. Malting decreased the level of flavan-4-ols in all samples, with the highest levels in the raw grains in G56, G99 and G22, and the highest levels in the malted sorghum in G22 followed by G56. Sorghums G99 and Dominator exhibited the greatest decreases in flavan-4-ol content (35% and 32%, respectively), while the flavan-4-ols of MR43 were reduced by only 16% (the minimum) after malting. Among all raw and malted sorghums, Buster had the lowest abundance of flavan-4-ols. Independent of malting process, grain sorghum with higher flavan-4-ol content might be more resistant to sooty stripe, sorghum midge, leaf anthracnose, and striga than in susceptible grains, as suggested by Dicko et al. [53].

Audilakshmi et al. [56] and Dicko et al. [49] reported that only 28% and 18% of sorghum cultivars, respectively, contained detectable amounts of flavan-4-ols, which is in contrast with the results for the Australian sorghum grains in the current study. Dicko et al. [49] found a 33% decrease in content of flavan-4-ols upon germination of sorghum, which is in rough agreement with the 25% decrease in flavan-4-ols in malted Australian red sorghum cultivars obtained here. The decrease of flavan-4-ol content in all cultivars upon malting may be related to the conversion of flavan-4-ols into other phenolic compounds such as anthocyanidins and flavan-3-ols. This process is accelerated by enzymes that are active during germination and is responsible for the biosynthesis of the other phenolics from flavan-4-ols as precursors [49,57].

### 2.5. Evaluation of Anthocyanins and Total Flavonoids

The anthocyanins are the most important group of natural water-soluble pigments that cause red, blue and purple colour in plant organs such as fruits, vegetables, flowers and grains. Market demand for foods containing anthocyanins has increased recently due to their potential health benefits as dietary antioxidants and also the range of colours they could provide as natural colourants in functional foods [58,59,60,61].

The grand mean contents for total anthocyanin in raw and malted sorghums were 6.98 and 16.82 abs/mL/g, respectively (Table 3). Total anthocyanin levels increased significantly about 2.4-fold in malted sorghum (*p <* 0.05). Sorghum grain harvested from Norwin had the greatest level of total anthocyanins, followed by Bellata (*p <* 0.05). 

The anthocyanin content of the Australian red sorghum cultivars ranged from 5.22 to 9.07 (abs/mL/g). This was mostly in the range 5.60 to 60.00 abs/mL/g for type I sorghums grown in USA [43]. Table 4 shows total anthocyanins in each of individual raw and malted sorghum grain cultivars. The anthocyanin level rose after malting in all samples, with raw G22 having the highest anthocyanin content followed by raw Eclipse. The highest anthocyanin levels in the malted grains were in MR43 and G22. The greatest increase (3.0-fold) in anthocyanin level was found in MR43 while this change was lowest (2.2-fold) in Bazley.

Malting the sorghum grains lead to an increase in anthocyanin content. Dicko et al. [49] reported no impact on anthocyanin content upon germination while Nwanguma and Eze [32] found an increase. The increase in the content of anthocyanin in germinated sorghum grain could be due to de novo synthesis and polymerization of the phenolics during germination [62].

Total flavonoids in the raw and malted red sorghum cultivars were compared (Table 3). The overall means for raw and malted sorghum were 1.24 and 1.09 mg CE/g, respectively. The total flavonoid level decreased significantly in malted sorghum grains by about 12% (*p <* 0.05). Bellatta had a significant higher level of total flavonoids at a value of 1.29 mg CE/g than Norwin and Yallaroi (*p <* 0.05).

The total flavonoid content decreased upon malting in each of the individual sorghum hybrids (Table 4). Raw sorghums Eclipse, Buster and MR43 had the highest levels of total flavonoids at values of 1.32, 1.29 and 1.29 mg CE/g, respectively, while Bazley had the lowest with 1.17 mg CE/g. The total flavonoid values for the Australian red sorghums were in a range reported for sorghum flour at a level of 0.50 to 6.81 mg CE/g by Herald et al. [63] and 1.16 mg CE/g by Afify et al. [64].

The highest flavonoid content among the malted samples was found in Eclipse and G22. The greatest decrease in flavonoid level upon malting was in Buster (24%) while this change was the least in G22 (2.5%).

The abundance of flavonoids was on average 3 to 10-fold higher than the total flavonoid content of wheat (0.38 mg CE/g), oat (0.41 mg CE/g) and buckwheat (0.15 mg CE/g) [65,66]. Afify et al. [64] and Gujral et al. [65] also reported a decline in the level of total flavonoids for soaked sorghum and kilned oat grain, respectively. The decrease in the level of total flavonoids might be due to their sensitivity to thermal processing or to conversion to other phenolic compounds during germination [62,66].

#### 3-Deoxyanthocyanins in Raw and Malted Sorghum Grains

Luteolinidin, apigeninidin, 5-methoxy-luteolinidin and 7-methoxyapigeninidin were identified here as major 3-deoxyanthocyanins in all raw and malted sorghum grains. Overall, malting significantly increased the levels of luteolinidin, apigeninidin, 5-methoxy-luteolinidin and 7-methoxy-apigeninidin (*p <* 0.05) (Table 5). The levels of luteolinidin and apigeninidin increased by an order of magnitude upon malting from 4.28 to 43.93 μg/g and from 4.43 to 37.89 μg/g, respectively. A similar trend was observed for the levels of 5-methoxy-luteolinidin and 7-methoxy-apigeninidin (Table 5). Among all the 3-deoxyanthocyanins, luteolinidin showed the greatest change in abundance after malting, increasing >10-fold, while 5-methoxy-luteolinidin showed the least change, but still increasing almost 6-fold (Table 5). These results are in contrast to those of Dicko et al. [49], in which no changes were detected in the level of 3-deoxyanthocyanins in 50 varieties of sorghum grains, but are consistent with the results of Nwanguma and Eze [32], in which an increase in the level of 3-deoxyanthocyanins was observed in four sorghum varieties.

The increase in the 3-deoxyanthocyanin content in all the Australian cultivars upon malting could be attributed to activation of enzymes during germination that convert other flavonoids to 3-deoxyanthocyanins by de novo synthesis [57]. One of the enzymes involved in the conversion of other phenolics to 3-deoxyanthocyanins is phenylalanine ammonia lyase (PAL), which has been detected in green shoots of sorghum [67]. Other enzymes that might be responsible for the conversion of other phenolics to flavonoids during germination are chalcone synthase, chalcone isomerase, flavone synthase and dihydroflavonol 4-reductase [68].

Both raw and malted Australian sorghum grains had significantly lower levels of anthocyanins compared with other pigmented cereal grains, such as blue/red/purple corn (225–965 μg/g). However, malted sorghum had higher levels of anthocyanin than pink corn (93 μg/g), purple wheat (13–139 μg/g) and blue barley (4 μg/g) [50,69].

Sorghum grain from three harvesting regions showed significant differences in the levels of 3-deoxyanthocyanins. Norwin had the highest levels of all detected 3-deoxyanthocyanins compared to grains from Yallaroi and Bellatta (*p <* 0.05). Sorghum grain from Yallaroi had the second highest levels of apigeninidin and 7-methoxy-apigeninidin while grains from Bellatta had the second highest level of luteolinidin and 5-methoxy-luteolinidin (Table 5).

The effects of cultivar (genotype) × process (malting) on sorghum grain 3-deoxyanthocyanins are shown in Table 6. The total 3-deoxyanthocyanin level increased in all sorghum cultivars upon malting; the greatest increase was 11.7-fold in G56 while the smallest increase was 5.9-fold in G22.

Considering each individual Australian sorghum cultivar, MR43 had the highest abundance of 3-deoxyanthocyanins, with 26.74 and 175.71 μg/g in raw and malted grain, respectively. The lowest levels of 3-deoxyanthocyanin were 13.37 μg/g in raw G56 and 115.65 μg/g in malted Bazley, respectively. Compared to previous studies, the results obtained here for raw grains are in agreement with reported levels of 3-deoxyanthocyanins in non-tannin red sorghums, in which levels ranged from 0 to 139.2 μg/g [70].

In other research, the levels of total 3-deoxyanthocyanins in tannin-free red sorghums were in a range of 14 to 187 μg/g [71]. This difference between the results of the current study and others could be due to effects of cultivar and plant growth environment. The level of 3-deoxyanthocyanins in sorghum grains is known to be a key indicator for resistance of the grain to biotic and abiotic stresses [53]; for example, in research on resistance of sorghum grain to fungal infection, 3-deoxyanthocyanins (mainly apigeninidin) were shown to play the major defensive role [72].

The 3-deoxyanthocyanin profile varied among the sorghum cultivars. In raw grains, the highest levels of luteolinidin and 5-methoxy-luteolinidin were observed in G22, with 6.62 and 8.12 μg/g, respectively, accounting for 59% of total 3-deoxyanthocyanins in this cultivar. The lowest levels were in Bazley, with 2.27 and 2.25 μg/g, respectively, which were 33% of total 3-deoxyanthocyanins.

The content of apigeninidin and 7-methoxyapigeninidin in raw grains was highest in MR43, with 6.47 and 8.84 μg/g, respectively, contributing 57.3% to the total 3-deoxyanthocyanins. Raw Dominator grain had the lowest levels of apigeninidin and 7-methoxyapigeninidin, together contributing 45.2% of the total 3-deoxyanthocyanins.

In malted grain, the highest levels of luteolinidin observed were 55.30 μg/g in Eclipse and 54.76 μg/g in MR43. The abundance of apigeninidin was highest in Buster (45.95 μg/g) among all malted sorghum grains (Table 6). Malted G22 and Buster had the highest level of 5-methoxyluteolinidin and 7-methoxyapigeninidin, with 32.16 and 54.86 μg/g, respectively (Table 6). 

Luteolinidin content increased upon malting by 7.5 to 13.4-fold while the increase for apigeninidin was 6.0 to 12.3-fold. Bazley and Dominator displayed the greatest increase in luteolinidin and apigeninidin levels upon malting while G22 and MR43 gave the smallest rise. The greatest changes in the levels of 5-methoxyluteolinidin and 7-methoxyapigeninidin were observed in G56, with 8.0 and 15.1-fold increases after malting, respectively. Sorghums G22 and MR43 showed the smallest increase in the content of these methoxylated compounds (Table 6).

Luteolinidin and apigeninidin in all Australian red sorghum grain cultivars accounted for about 41.7% (G99) to 55.7% (G56) of the total 3-deoxyanthocyanins. These results are in agreement with those reported for the levels of these compounds in black sorghum grain analyzed by Awika et al. [73]. In the present study, G22 and MR43 had the highest levels of luteolinidin while MR43 and G99 had the highest levels of apigeninidin. In most sorghum cultivars, including G22, G99, MR43, Buster and Bazley, methoxylated derivatives (5-methoxyluteolinidin and 7-methoxyapigeninidin) were in greater abundance than their non-methoxylated counterparts. 

The 3-deoxyanthocyanin profiles showed different proportions of these compounds among the Australian grain sorghum cultivars. Apigeninidin and 7-methoxyapigeninidin contributed the main 3-deoxyanthocyanins in G99, MR43, Buster and Bazley, while G22 had higher combined levels of luteolinidin and 5-methoxyluteolinidin. G56, Dominator and Eclipse had almost the same content of luteolinidin, 5-methoxyluteolinidin, apigeninidin and 7-methoxyapigeninidin (Table 6). Luteolinidin and, to a lesser extent, apigeninidin, have been shown to suppress the viability of cancer cells in culture more than other anthocyanins such as cyanidin and pelargonidin [74].

Various factors could affect the production of 3-deoxyanthocyanins in sorghum grain. Dykes [43] reported that synthesis of 3-deoxyanthocyanins might be a plant reaction to environment such as sunlight, although genotype could be another determinant for the synthesis of 3-deoxyanthocyanins [44,75,76,77].

## 3. Materials and Methods

Commercial red sorghum hybrids Bazley, Buster, Dominator, Eclipse, G22, G56, G99 and MR43 were grown over the 2010–2011 season in three established sorghum-growing regions: Yallaroi and Bellata in Northern NSW and Norwin in Southern Qld. The minimum and maximum temperature and precipitation obtained during the grain-filling period (mid-January to mid-February) in 2011 from the Australian Bureau of Meteorology are presented in Table 7 [78].

Grains were harvested at maturity, air-dried and cleaned. Glumes were then removed and the samples sieved through a 3.0 mm screen. All grains used for analysis were milled to a fine flour using a cyclone sample mill (UD Corporation, Boulder, CO, USA) and sieved through a 0.5-mm screen.

### 3.1. Grain Malting

Grains of the eight sorghum hybrids were malted simultaneously. The three steps of the malting process—steeping, germination and kilning—were performed in a single unit using a Newmalt Micromalter (Joe White Malting Systems, Adelaide, Australia). Grains were steeped and germinated at 17 °C and kilned at 50–85 °C in the micromalter [79]. The malting program is presented in detail in Table 8.

For protection from mould growth during germination, the grains were immersed in 10% NaClO and then rinsed thoroughly in water before malting. The micromalter had 16 germination boxes, each of which was loaded with 400 g (dry basis) sorghum grain and rotated every 6 h to avoid rootlet and acrospire meshing. At the beginning of the germination cycle (after 24 h), 2560 μg gibberellic acid (GA) in 800 mL deionised water was distributed equally among all the boxes. To restore grain moisture levels to 46%, deionised water was added at 48- and 72-h time-points during the germination stage. Cultivars from each site were randomised between germination boxes. At the end of the malting process, samples were cleaned of excess root and shoot growth, labelled and stored at 4 °C.

### 3.2. Chemical Reagents 

Folin-Ciocalteu reagent, gallic acid, catechin hydrate, Trolox, ethanolamine, were obtained from Sigma-Aldrich (St. Louis, MO, USA). Luteolinidin chloride, apigeninidin chloride and 7-methoxy-apigeninidin were obtained from ChromaDex (Santa Ana, CA, USA). *sec*-Butanol was reagent grade and all other solvents were HPLC grade.

### 3.3. Colour Measurement

The colour of the sorghum grains was determined using a CR-300 Minolta Chroma Meter (Minolta Co., Ltd., Osaka, Japan). This measurement is quantified by the Hunterlab system giving L*, a* and b* parameters. The maximum L* is 100 and represents white. The minimum L* is zero and represents black. The a* and b* values have no specific numerical limits. Positive a* is red; negative a* is green. Positive b* is yellow; negative b* is blue.

### 3.4. Total Phenolic Content

Total phenolic compounds were measured using the modified Folin-Ciocalteu method of Kaluza et al. [80]. An aliquot (0.1 mL) of the extract was added to 1.1 mL of water, and reacted with 0.9 mL 0.5 M ethanolamine and 0.4 mL of Folin-Ciocalteu reagent. The solution was allowed to stand for 20 min at ambient temperature in the dark and the absorbance read at 600 nm. Gallic acid was used as a standard.

### 3.5. Condensed Tannin

The presence or absence of a pigmented testa was determined by the Clorox bleach test [37]. Sorghum grains (15 g) were mixed with 7.5 g KOH and 70 mL NaOCl solution (bleach) with constant stirring at 60 °C for 7 min, rinsed and washed with cold water. The grains that turned white or yellow were classified as type I sorghums, while a black colour indicated the presence of condensed tannin (type II or III). 

Condensed tannins were determined by the modified vanillin/HCl assay described by Price et al. [81]. Milled sample (0.2 g) was mixed with 10 mL of 1% HCl in MeOH (*v*/*v*) for 20 min while shaking at low speed (KS501 D shaker, IKA Labortechnik, Staufen, Germany). The mixture was then centrifuged at 3000× *g* for 10 min. Both the supernatant and reagents were at 30 °C at the start of the assays. An aliquot (1 mL) of the extract was transferred to a capped test tube and, within 1 min, 5 mL of vanillin reagent was added and the mixture kept in a water bath at 30 °C for 20 min. The absorbance was read at 500 nm. A blank was prepared by replacing the vanillin reagent with 4% HCl in MeOH. Catechin was applied as a standard for the condensed tannin assay.

### 3.6. Flavan-4-ols, Total Anthocyanins and Total Flavonoids

The content of flavan-4-ols was measured using the modified method described by Gous [82]. Briefly, 1 mL of sample extract was reacted with 5 mL of HCl-butanol reagent, made by dissolving 0.0616 g of FeSO_4_·7H_2_O in 5% HCl in sec-butanol (*v*/*v*). The reaction mixture was kept for 1 h at ambient temperature and the absorbance measured at 550 nm. The result was expressed as absorbance per milliliter per gram dry weight of sample (abs/mL/g).

Total anthocyanin was determined according to the method of Fuleki and Francis [83]. One portion of sample (acidified methanolic extract) was diluted by two portions of extraction solvent and allowed to stand for 2 h in dark at room temperature. The absorbance was then read at 485 nm and reported as absorbance per millilitre (Abs/mL) per gram of dry weight sample.

Total flavonoids was measured using the assay described by Afify et al. [64]. Ground sorghum grain (1 g) was extracted using 10 mL of 80% MeOH. After shaking for 2 h, 0.4 mL of the sorghum extract was mixed with 4 mL of distilled water and 0.3 mL of 5% NaNO_2_ and left for 5 min. An aliquot (0.3 mL) of 10% AlCl_3_ was added to the mixture, which was incubated for 6 min and then mixed with 2 mL of 1 M NaOH and the total volume made up to 10 mL with distilled water. The absorbance was read at 510 nm and the result expressed as μg catechin equivalent/g dry sample (μg/g).

### 3.7. Quantification and Identification of 3-Deoxyanthocyanins Using HPLC 

For 3-deoxyanthocyanins, ground samples of whole grain sorghum flour (1 g) were extracted in 10 mL of 1% HCl/methanol (*v*/*v*) for 2 h in a shaker. The extracts were centrifuged at 2790 *g* for 10 min and decanted [71]. All extracts were immediately filtered using a 0.45-μm nylon membrane filter (Grace Davison Discovery Science, Columbia, IL, USA) prior to HPLC analyses.

Extracts were analyzed according to the methods of Dykes et al. [70] applying reversed phase HPLC (Agilent Technologies 1200 series, Santa Clara, CA, USA) connected to a photodiode array detector (PDA), with slight modification. Sorghum 3-deoxyanthocyanins, were separated using a Hypersil BDS C18 column (250 mm × 4.6 mm, i.d. 5 μm) from Shandon Southern Instruments Ltd. (Runcorn, UK). The sample extract was injected in a volume of 20 μL. The mobile phase consisted of high-purity water:formic acid 96:4 (*v*/*v*) (Solvent A) and acetonitrile (Solvent B). The solvent flow rate was 1.0 mL/min. The 3-deoxyanthocyanins were separated using the following gradient: 0–4 min, 12% B; 4–25 min, 20% B; 25–40 min, 40% B, and detected at 485 nm. 

Identification of sorghum 3-deoxyanthocyanins, was performed on the basis of retention times of commercial standards, and on LC-MS data. Quantification of each compound was achieved by comparing peak areas with those in a standard curve for each authentic standard. Molecular weight correction factors [84] were used to quantify 5-methoxy-luteolinidin. Data were collected and processed using Chemstation software (Agilent Technologies).

### 3.8. Statistical Analysis

Data were subjected to analysis of variance (ANOVA) procedures appropriate for a completely randomized design with a factorial arrangement (8 sorghum cultivars × 2 processes (unmalted and malted) × 3 regions (Yallaroi, Norwin and Bellatta) using the general linear model procedures of the Statistical Analysis System (SAS 9.4 package, SAS Institute, Cary, NC, USA). After ANOVA, all data were tested for normality and a transformation of the data was found not to be necessary. The least-squares mean (LS Means) test was used to assess any significant differences among treatments. A *p*-value less than 0.05 was considered as the significance level for mean comparisons.

## 4. Conclusions

Raw and malted grains of a wide variety of Australian sorghum grain cultivars were analyzed for total phenolics, condensed tannins, flavan-4-ols, and total anthocyanins. The 3-deoxyanthocyanin profile was evaluated for all the sorghum cultivars. 

Using a bleach test and a vanillin assay, condensed tannin was found to be absent from grains of the Australian sorghum cultivars, which showed that they are indeed type I sorghums. 

On average for each cultivar from the three different regions, the highest total phenolic content, flavan-4-ols, total flavonoids and antioxidant activities in all cultivars were found in Eclipse and G56, while the lowest levels were in Buster, Bazley and Dominator. Previous work showed that the group of cultivars suitable for porridge (one of the principle sorghum-based foods in the developing world) have on average a low level of total phenolic compounds, flavan-4-ols, anthocyanins, as well as low antioxidant activity [49]. Raw grains of cultivars MR43, G22 and G99 had the highest levels of 3-deoxyanthocyanin, mainly apigeninidin. The grain content of these compounds, particularly apigenidinin, could be a valuable indicator for breeders to select for grain resistance to fungi such as *Colletrichum graminicola*, *Fusarium oxysporum*, *Gibberelle zeae* and *Gliocladium roseum* [72] and also to select sorghum cultivars with suitable content of healthy phenolic compounds. 

The levels of total phenolics, flavan-4-ols, total flavonoids in all the Australian sorghum cultivars decreased upon malting, whereas the level of total anthocyanins increased. With respect to the 3-deoxyanthocyanins, malting resulted in a significant increase in the levels of luteolinidin, apigeninidin, 5-methoxyluteolinidin and 7-methoxyapigeninidin. Sorghum MR43 in malted form had the highest level of 3-deoxyanthocyanins. Sorghum cultivars that contain a high content of flavonoids such as 3-deoxy anthocyanins, flavones and flavanones before and after malting could be a good source of the reddish colour desired by sorghum beverage manufacturers, while a low level of flavonoids would be best for couscous and porridge preparation [49].

The sensory properties of tannin-free sorghum grains may be superior to tannin-containing sorghums but this needs more investigation. Thus, a focus for future research should be on producing sorghum-based functional foods in which raw sorghum grains, malted grains or malt extracts are used as an ingredient. Evaluation of the possible in vivo antioxidant activity of malted sorghum needs to be studied as the knowledge gained could give valuable insights into the potential health effects. This is because malting led to significant increases in the levels of specific phenolic compounds such as 3-deoxyanthocyanins, which are associated with better growth inhibition of human leukemia HL-60 and hepatoma hep G2 cell lines than common anthocyanins [74]. Such information would help the health and food industry to select suitable sorghum cultivars from which to make functional foods. 

## Figures and Tables

**Table 1:** Overall mean comparisons for colour of Australian-grown sorghums between processes (raw versus malted), regions and cultivars. Values are means ± SD.

Treatment			L*	a*	b*
Process	Region	Cultivar			
Raw			43.26 ± 2.08 ^a^	16.77 ± 1.21 ^a^	16.99 ± 1.87 ^a^
Malted			40.82 ± 2.08 ^b^	15.10 ± 1.18 ^b^	14.44 ± 2.12 ^b^
	Yallaroi		43.56 ± 2.06 ^a^	15.96 ± 1.18 ^b^	17.40 ± 1.66 ^a^
	Norwin		39.89 ± 1.44 ^c^	16.20 ± 1.69 ^a^	13.77 ± 2.10 ^b^
	Bellatta		42.67 ± 2.47 ^b^	15.66 ± 1.44 ^c^	15.97 ± 1.76 ^c^
		G22	42.57 ± 1.73 ^c^	17.33 ± 1.12 ^a^	17.43 ± 2.09 ^a^
		G56	40.76 ± 2.00 ^e^	16.65 ± 1.24 ^b^	15.00 ± 2.43 ^f^
		G99	41.60 ± 1.83 ^d^	15.96 ± 1.23 ^d^	15.60 ± 2.38 ^d^
		Dominator	41.06 ± 1.53 ^e^	16.46 ± 1.68 ^c^	15.23 ± 1.75 ^e^
		Eclipse	40.96 ± 1.79 ^e^	15.60 ± 1.21 ^e^	14.36 ± 2.02 ^g^
		MR43	42.65 ± 1.89 ^b,c^	15.37 ± 1.45 ^f^	15.96 ± 1.66 ^c^
		Buster	43.78 ± 3.64 ^a^	14.58 ± 0.95 ^g^	16.44 ± 2.90 ^b^
		Bazley	42.95 ± 3.78 ^b^	15.57 ± 1.06 ^e^	15.70 ± 2.73 ^d^

Means with the same letter in a column are not significantly different (*p >* 0.05).

**Table 2:** Mean comparison of sorghum cultivar and process interaction on grain colour. Values are means ± SD (*n* = 6).

Cultivar	Process	L*	a*	b*
G22	Raw	43.64 ± 0.93 ^c^	18.26 ± 0.32 ^a^	18.73 ± 1.06 ^a^
	Malted	41.51 ± 1.73 ^g,h^	16.40 ± 0.76 ^e^	16.12 ± 2.09 ^e^
G56	Raw	41.88 ± 2.11 ^f,g^	17.42 ± 0.59 ^b^	16.13 ± 2.25 ^e^
	Malted	39.64 ± 1.16 ^j^	15.88 ± 1.26 ^g^	13.87 ± 2.21 ^j^
G99	Raw	42.79 ± 1.65 ^d^	15.44 ± 1.56 ^h^	16.81 ± 1.97 ^d^
	Malted	40.41 ± 1.11 ^i^	16.48 ± 0.49 ^d^	14.40 ± 2.26 ^i^
Dominator	Raw	42.46 ± 0.48^d,e^	17.44 ± 1.73 ^b^	16.77 ± 0.60 ^d^
	Malted	39.66 ± 0.45 ^j^	15.48 ± 0.97 ^h^	13.69 ± 0.84 ^k^
Eclipse	Raw	42.19 ± 1.50 ^e,f^	16.62 ± 0.83 ^c^	15.89 ± 0.93 ^f^
	Malted	39.74 ± 1.11 ^j^	14.57 ± 0.40 ^k^	12.84 ± 1.60 ^l^
MR43	Raw	43.99 ± 1.70 ^b,c^	16.25 ± 1.37 ^f^	17.35 ± 0.73 ^c^
	Malted	41.31 ± 0.79 ^h^	14.50 ± 0.96 ^l^	14.57 ± 0.96 ^h^
Buster	Raw	44.94 ± 3.75 ^a^	15.33 ± 0.72 ^i^	17.56 ± 2.60 ^b^
	Malted	42.63 ± 3.43 ^d,e^	13.83 ± 0.32 ^m^	15.32 ± 2.96 ^g^
Bazley	Raw	44.19 ± 4.15 ^b^	16.39 ± 0.48 ^e^	16.70 ± 2.76 ^d^
	Malted	41.71 ± 3.24 ^f,g,h^	14.74 ± 0.77 ^j^	14.70 ± 2.54 ^h^

Means with the same letter in a column are not significantly different (*p >* 0.05).

**Table 3:** Overall comparison of phenolics and antioxidant activity in raw versus malted grains, regions and cultivars of Australian sorghums. Values are means ± SD.

Treatment			TPC	Tannin	Flavan-4-ols	T-Anthocyanins	T-Flavonoids
Process	Region	Cultivar					
Raw			2.77 ± 0.38 ^a^	0.86 ± 0.26 ^a^	2.98 ± 0.57 ^a^	6.78 ± 2.26 ^b^	1.24 ± 0.19 ^a^
Malted			2.48 ± 0.25 ^b^	0.63 ± 0.16 ^b^	2.23 ± 0.60 ^b^	16.82 ± 6.78 ^a^	1.09 ± 0.15 ^b^
	Yallaroi		2.79 ± 0.32 ^a^	0.78 ± 0.28 ^a^	2.57 ± 1.02 ^b^	9.18 ± 3.86 ^c^	1.05 ± 0.12 ^c^
	Norwin		2.72 ± 0.30 ^a^	0.79 ± 0.24 ^a^	2.54 ± 0.62 ^b^	16.40 ± 9.31 ^a^	1.16 ± 0.16 ^b^
	Bellatta		2.37 ± 0.29 ^b^	0.67 ± 0.17 ^b^	2.70 ± 0.13 ^a^	9.82 ± 4.34 ^b^	1.29 ± 0.20 ^a^
		G22	2.66 ± 0.42 ^b,c^	0.82 ± 0.35 ^a,b^	2.86 ± 0.48 ^a^	14.37 ± 8.42 ^a^	1.19 ± 0.22 ^c^
		G56	2.80 ± 0.28 ^a^	0.80 ± 0.31 ^a,b^	2.89 ± 0.90 ^a^	12.50 ± 6.41 ^d^	1.16 ± 0.22 ^d^
		G99	2.61 ± 0.41 ^c,d^	0.69 ± 0.19 ^b,c^	2.66 ± 0.87 ^b^	12.33 ± 6.08 ^d^	1.76 ± 1.15 ^d^
		Dominator	2.49 ± 0.39 ^d,e^	0.75 ± 0.27 ^a,b^	2.56 ± 0.67 ^c^	10.22 ± 6.28 ^e^	1.16 ± 0.20 ^d^
		Eclipse	2.77 ± 0.32 ^a,b^	0.84 ± 0.18 ^a^	2.71 ± 0.63 ^b^	12.93 ± 7.46 ^c^	1.25 ± 0.14 ^a^
		MR43	2.67 ± 0.28 ^b,c^	0.72 ± 0.20 ^a,b,c^	2.65 ± 0.66 ^b^	13.46 ± 9.92 ^b^	2.86 ± 1.21 ^b^
		Buster	2.43 ± 0.35 ^e^	0.62 ± 0.15 ^c^	2.16 ± 0.56 ^e^	10.07 ± 6.56 ^e^	1.14 ± 0.25 ^e^
		Bazley	2.60 ± 0.27 ^d,c^	0.72 ± 0.21 ^a,b,c^	2.36 ± 0.52 ^d^	8.52 ± 4.01 ^f^	1.08 ± 0.22 ^f^

TPC (mg GAE/g dry basis); tannin (mg CE/g dry basis); flavan-4-ols and total anthocyanins (abs/mL/g dry basis); total flavonoids (mg CE/g dry basis). GAE = Gallic acid equivalents; TE = Trolox equivalents; CE = Catechin equivalents. Means with the same letter in a column are not significantly different (*p >* 0.05).

**Table 4:** Mean comparison of grain phenolics and antioxidant activity among Australian sorghum cultivars. Values are means ± SD (*n* = 6).

Cultivar	Process	TPC	Tannin	Flavan-4-ols	T-Anthocyanins	T-Flavonoids
G22	Raw	2.80 ± 0.58 ^a,b,c^	1.02 ± 0.38 ^a^	3.11 ± 0.58 ^b,c^	9.07 ± 1.83 ^h^	1.21 ± 0.15 ^e^
	Malted	2.52 ± 0.12 ^e,f,g^	0.61 ± 0.16 ^d,e^	2.61 ± 0.13 ^e,f^	19.66 ± 9.23 ^b^	1.18 ± 0.29 ^f,g^
G56	Raw	2.85 ± 0.33 ^a,b^	0.91 ± 0.38 ^a,b^	3.27 ± 1.01 ^a^	7.16 ± 1.43 ^k,j^	1.22 ± 0.18 ^d^
	Malted	2.75 ± 0.23 ^b,c,d^	0.68 ± 0.21 ^c,d,e^	2.52 ± 0.63 ^f,g^	17.84 ± 4.47 ^c^	1.09 ± 0.09 ^i^
G99	Raw	2.84 ± 0.45 ^a,b^	0.83 ± 0.19 ^b,c^	3.22 ± 0.83 ^a,b^	7.50 ± 1.71 ^j^	1.22 ± 0.16 ^d,e^
	Malted	2.37 ± 0.19 ^g,h^	0.55 ± 0.01 ^e^	2.09 ± 0.48 ^i^	17.16 ± 4.73 ^d^	1.09 ± 0.06 ^i^
Dominator	Raw	2.59 ± 0.46 ^d,e,f^	0.90 ± 0.30 ^a,b^	3.05 ± 0.35 ^c^	5.80 ± 1.76 ^l^	1.25 ± 0.22 ^c^
	Malted	2.40 ± 0.33 ^g^	0.61 ± 0.16 ^d,e^	2.07 ± 0.54 ^i^	14.65 ± 6.07 ^f^	1.07 ± 0.15 ^j^
Eclipse	Raw	2.97 ± 0.35 ^a^	0.90 ± 0.16 ^a,b^	3.10 ± 0.36 ^b,c^	7.98 ± 3.04 ^i^	1.32 ± 0.11 ^a^
	Malted	2.58 ± 0.15 ^d,e,f^	0.77 ± 0.20 ^b,c,d^	2.32 ± 0.62 ^h^	17.89 ± 7.37 ^c^	1.19 ± 0.15 ^f^
MR43	Raw	2.75 ± 0.34 ^b,c,d^	0.76 ± 0.21 ^b,c,d^	2.87 ± 0.33 ^d^	6.75 ± 2.91 ^k^	1.29 ± 0.16 ^b^
	Malted	2.59 ± 0.20 ^d,e,f^	0.68 ± 0.21 ^c,d,e^	2.41 ± 0.86 ^g,h^	20.18 ± 10.00 ^a^	1.13 ± 0.17 ^h^
Buster	Raw	2.65 ± 0.34 ^c,d,e^	0.69 ± 0.20 ^c,d,e^	2.50 ± 0.18 ^f,g^	4.78 ± 1.06 ^m^	1.29 ± 0.27 ^b^
	Malted	2.21 ± 0.19 ^h^	0.55 ± 0.00 ^e^	1.82 ± 0.61 ^j^	15.35 ± 5.15 ^e^	0.98 ± 0.08 ^l^
Bazley	Raw	2.75 ± 0.23 ^b,c,d^	0.83 ± 0.21 ^b,c^	2.70 ± 0.14 ^e^	5.22 ± 0.39 ^m^	1.17 ± 0.30 ^g^
	Malted	2.45 ± 0.23 ^g,f^	0.61 ± 0.17 ^d,e^	2.03 ± 0.56 ^i^	11.82 ± 3.04 ^g^	1.00 ± 0.03 ^k^

TPC (mg GAE/g dry basis); tannin (mg CE/g dry basis); flavan-4-ols and total anthocyanins (abs/mL/g dry basis); total flavonoids (mg CE/g dry basis). GAE = Gallic acid equivalents; TE = Trolox equivalents; CE = Catechin equivalents. Means with the same letter in a column are not significantly different (*p* > 0.05).

**Table 5:** Overall comparison of 3-deoxyanthocyanin abundance (μg/g dry basis) in raw versus malted, regions and grain cultivars of Australian sorghums. Values are means ± SD.

Treatment			Luteolinidin	Apigeninidin	5-Methoxy-luteolinidin	7-Methoxy-apigeninidin
Process	Region	Cultivar				
Raw			4.28 ± 4.24 ^b^	4.43 ± 3.20 ^b^	4.35 ± 4.19 ^b^	5.95 ± 5.34 ^b^
Malted			43.93 ± 33.13 ^a^	37.89 ± 12.27 ^a^	25.71 ± 23.08 ^a^	46.09 ± 19.78 ^a^
	Yallaroi		12.58 ± 11.95 ^c^	20.05 ± 18.91 ^b^	5.71 ± 4.59 ^c^	20.42 ± 19.73 ^b^
	Norwin		45.16 ± 42.88 ^a^	25.73 ± 20.69 ^a^	31.99 ± 25.95 ^a^	39.41 ± 30.78 ^a^
	Bellatta		14.58 ± 15.44 ^b^	17.69 ± 17.01 ^c^	7.39 ± 6.74 ^b^	18.22 ± 16.32 ^c^
		G22	28.09 ± 33.06 ^b^	14.87 ± 12.36 ^e^	20.14 ± 23.12 ^a^	22.47 ± 22.57 ^c^
		G56	23.31 ± 22.84 ^c,d^	24.27 ± 24.44 ^a,b^	13.20 ± 16.94 ^d,e^	24.04 ± 29.28 ^c^
		G99	24.27 ± 22.40 ^c^	22.61 ± 20.67 ^c^	16.07 ± 16.00 ^c^	30.15 ± 30.58 ^a^
		Dominator	22.00 ± 29.73 ^d^	19.08 ± 17.47 ^d^	14.18 ± 20.07 ^d^	23.37 ± 22.49 ^c^
		Eclipse	30.50 ± 46.95 ^a^	22.09 ± 18.98 ^c^	17.05 ± 25.81 ^c^	27.16 ± 24.73 ^b^
		MR43	30.48 ± 43.66 ^a^	22.70 ± 17.37 ^b,c^	18.20 ± 26.41 ^b^	29.83 ± 23.36 ^a^
		Buster	17.88 ± 21.11 ^e^	25.32 ± 25.25 ^a^	12.28 ± 16.64 ^e^	30.24 ± 8.90 ^a^
		Bazley	16.32 ± 20.48 ^e^	18.32 ± 15.97 ^d^	9.14 ± 11.37 ^f^	20.89 ± 16.84 ^d^

Means with the same letter in a column are not significantly different (*p >* 0.05).

**Table 6:** Mean comparison for 3-deoxyanthocyanins (μg/g dry basis) in raw and malted grains of Australian sorghum cultivars. Values are means ± SD (*n* = 6).

Cultivar	Process	Luteolinidin	Apigeninidin	5-Methoxyluteolinidin	7-Methoxyapigeninidin
G22	Raw	6.62 ± 6.07 ^g^	3.90 ± 3.98 ^f,g^	8.12 ± 6.55 ^f^	6.36 ± 6.38 ^h^
	Malted	49.56 ± 35.53 ^b^	25.85 ± 5.56 ^d^	32.16 ± 28.04 ^a^	38.57 ± 21.40 ^e^
G56	Raw	3.65 ± 0.60 ^h,i^	3.79 ± 3.03 ^f,g^	2.94 ± 0.73 ^j,h,i^	2.99 ± 2.02 ^j^
	Malted	42.97 ± 14.81 ^c,d^	44.74 ± 17.28 ^a^	23.46 ± 19.44 ^c^	45.08 ± 28.62 ^d^
G99	Raw	3.67 ± 1.55 ^h,i^	5.41 ± 5.99 ^e,f^	3.98 ± 2.57 ^g,h,i^	8.74 ± 10.68 ^g^
	Malted	44.86 ± 9.16 ^c^	39.80 ± 13.97 ^b^	28.17 ± 14.34 ^b^	51.55 ± 29.05 ^b^
Dominator	Raw	3.63 ± 2.94 ^h,i^	2.87 ± 0.85 ^g^	4.23 ± 3.37 ^g,h^	3.60 ± 1.22 ^I,j^
	Malted	40.37 ± 33.56 ^d^	35.30 ± 6.28 ^c^	24.12 ± 25.24 ^c^	43.13 ± 13.17 ^d^
Eclipse	Raw	5.70 ± 6.77 ^g,h^	4.72 ± 2.04 ^e,f,g^	5.34 ± 6.11 ^g^	5.83 ± 2.27 ^h^
	Malted	55.30 ± 57.68 ^a^	39.46 ± 8.02 ^b^	28.77 ± 33.14 ^b^	48.48 ± 15.68 ^c^
MR43	Raw	6.21 ± 6.92 ^g,h^	6.47 ± 3.05 ^e^	5.22 ± 5.48 ^g^	8.84 ± 6.07 ^g^
	Malted	54.76 ± 52.26 ^a^	38.93 ± 4.69 ^b^	31.20 ± 33.18 ^a^	50.82 ± 10.34 ^b^
Buster	Raw	2.49 ± 1.76 ^i^	4.68 ± 2.42 ^e,f,g^	2.77 ± 2.30 ^i,j^	5.62 ± 3.95 ^h,i^
	Malted	33.28 ± 20.23 ^e^	45.95 ± 19.39 ^a^	21.78 ± 19.68 ^d^	54.86 ± 24.94 ^a^
Bazley	Raw	2.27 ± 0.88 ^i^	3.57 ± 2.11 ^f,g^	2.25 ± 0.99 ^j^	5.57 ± 3.31 ^h,i^
	Malted	30.36 ± 21.18 ^f^	33.06 ± 5.93 ^c^	16.03 ± 13.01 ^e^	36.20 ± 7.08 ^f^

Means with the same letter in a column are not significantly different (*p >* 0.05).

**Table 7:** Sorghum growing regions mean precipitation and temperature during grain filling.

Region	Mean Temperature (°C)		Precipitation (mm)
	Min	Max	
Norwin	18	29.8	34.3
Yallaroi	21	34.5	44.2
Bellata	20.8	34.5	57.4

**Table 8:** Joe White Malting Systems Newmalt micromalter standard program.

Stage	Stage	Temperature (°C)	Time (h)
Steeping	Water	17	8:00
	Air rest	17	10:00
	Water	17	2:00
Germination	Water bed	17	96:00
Kilning	Air	50	6:00
		55	2:00
		60	2:00
		65	1:30
		70	1:30
		75	3:30
		85	3:30
